# Social workers and acquired brain injury: A systematic review of the current evidence-base

**DOI:** 10.1371/journal.pone.0292128

**Published:** 2023-11-10

**Authors:** Mark A. Linden, Mark Holloway, Claire Cooper, Akudo Amadiegwu, Caroline Bald, Michael Clark, Andy Mantell, Alyson Norman, Andrew Bateman

**Affiliations:** 1 School of Nursing and Midwifery, Queen’s University Belfast, Belfast, United Kingdom; 2 Head First Assessment Rehabilitation and Case Management LLP, Cranbrook, United Kingdom; 3 School of Health and Social Care, University of Essex, Colchester, United Kingdom; 4 Care Policy and Evaluation Centre, London School of Economics & Political Science, London, United Kingdom; 5 The Brain Injury Social Work Group, Maidstone, United Kingdom; 6 School of Psychology, University of Plymouth, Plymouth, United Kingdom; University of Saskatchewan, CANADA

## Abstract

Social work plays an important role in the assessment and treatment of people with acquired brain injury. Acquired brain injury is a complex and highly prevalent condition which can impact on cognitive, emotional and social domains. As acquired brain injury is a hidden disability it can be misdiagnosed or classified as another condition entirely. We sought to systematically explore the evidence base to examine how social workers have been prepared to work with their clients with brain injury. Employing six electronic databases (Social Policy & Practice, Web of Science, Scopus, PubMed, PsycINFO, CINAHL Plus) we reviewed 1071 papers. After applying eligibility criteria 17 papers were included in this review. We utilised standardised data extraction and quality appraisal tools to assess all included papers. Following appraisal, 9 papers were judged as possessing high methodological quality whilst 8 were judged as medium. Employing narrative synthesis, we identified four themes which captured the key findings of these papers. Themes were named as (i) advocacy and social work (ii) training and multidisciplinary team working (iii) inclusion of social networks and (iv) societal barriers. In order to meet their statutory responsibilities to practice safely, social workers must receive training in how to identify ABI and develop understanding of its consequences and subsequent need for provision. Social workers are also in a unique position to advocate for their clients and should make every effort to ensure their needs are met.

## Introduction

An estimated 1.3 million people in the UK live with an Acquired Brain Injury (ABI) related disability [1]. Acquired Brain Injury is defined as any injury to the brain which has occurred since birth and can result from traumatic brain injuries (TBI), such as road traffic accidents, assaults, falls, illness, or from diseases of the brain, or lack of oxygen to the brain (hypoxia) [2, 3].

The impact of ABI varies considerably from person to person but can include physical, sensory, cognitive and executive functioning impairments, changes to behaviour, personality, emotional state, and difficulties with communication [4–8]. These difficulties can negatively affect functioning and community integration, employment prospects, and parenting abilities, and people with an ABI are notably over-represented in prisoner and homelessness populations, and the condition is implicated in intimate partner violence, both as victim and perpetrator [9–15]. The impact of ABI is noted to not be simply confined to that of the person with the injury, but to have a significant impact on family and friends [16–18].

Bearing in mind this panoply of difficulties with functioning following an ABI, it is therefore to be expected that the condition forms part of the day-to-day work of a Social Worker, either directly or indirectly [19]. ABI is however noted to be relatively poorly researched by academic social work [20] and criticisms of the profession’s response to the needs of individuals and families affected by the condition have grown [21–24].

This systematic review sought to identify and synthesise the evidence base on social workers interactions with survivors of ABI. This included research where social workers were participants in research and where guidelines or guidance for social workers were offered in regard to ABI.

## Methods

### Information sources

A total of six electronic databases were searched which included articles in the fields of medicine and social sciences. These included Social Care Online, Web of Science, Scopus, PubMed, PsycINFO, CINAHL Plus. Initial explorations of literature on the topic aided the identification of key words. These were then extracted from the relevant articles and reviewed by the team for inclusion in the search strategy. The reference lists of all included articles were also examined to determine if any relevant articles had been missed. The Preferred Reporting Items for Systematic reviews and Meta-Analyses (PRISMA) guidance was followed in conducting and reporting this review [25].

### Search strategy

Key words identified from the literature, together with search terms employed in previous systematic reviews [26, 27] were combined with MeSH headings to produce a list of search terms for this review. We based these around the key concepts of interest which were ‘social work’ and ‘acquired brain injury’ (see Table 1 for search terms). Our search strategy was then reviewed by a specialist subject librarian. The Boolean operator OR was used to include all search terms under the same concept whilst the operator AND was used to combine concepts. Searches were conducted in August 2022.

**Table 1:** Search terms employed in this review.

Concepts	Social work	Acquired Brain Injury
Search terms	Social Workers (MeSH) Social Work (MeSH) Social Casework	Brain Injuries (MeSH) Brain Injury Head injuries (MeSH) Acquired Brain Injury Craniocerebral Trauma (MeSH) Cerebrovascular Trauma (MeSH) Brain Edema (MeSH) Brain Swelling Cerebral Edema Glasgow Coma Scale (MeSH) Glasgow Outcome Scale (MeSH) Unconsciousness (MeSH) Pneumocephalus (MeSH) Epilepsy, Post-Traumatic (MeSH) Cerebral Hemorrhage (MeSH) Brain Damage Head or crani* or cerebr* or brain* or skull* or intercran* Injur* or trauma* or damage* or wound* or haemorrhag* or hemorrhag*

### Eligibility criteria

As we sought to identify only the most recent developments in this field we limited our searches to articles published between 2012–2022. These articles had to have a primary focus on social work or social workers and had to include information about their working with ABI. If an article included ABI or social work as a component of other disability types or professions we applied a preponderance rule i.e. if more than 50% of included disabilities were ABI or 50% of professions were social work the article was included. We did not limit articles based on study design, country of origin or whether these presented empirical data. As such, discussion articles alongside reviews and guidelines were included if they met the inclusion criteria. Due to resource constraints we limited our searches to articles written in English.

### Selection process

**Fig 1:**
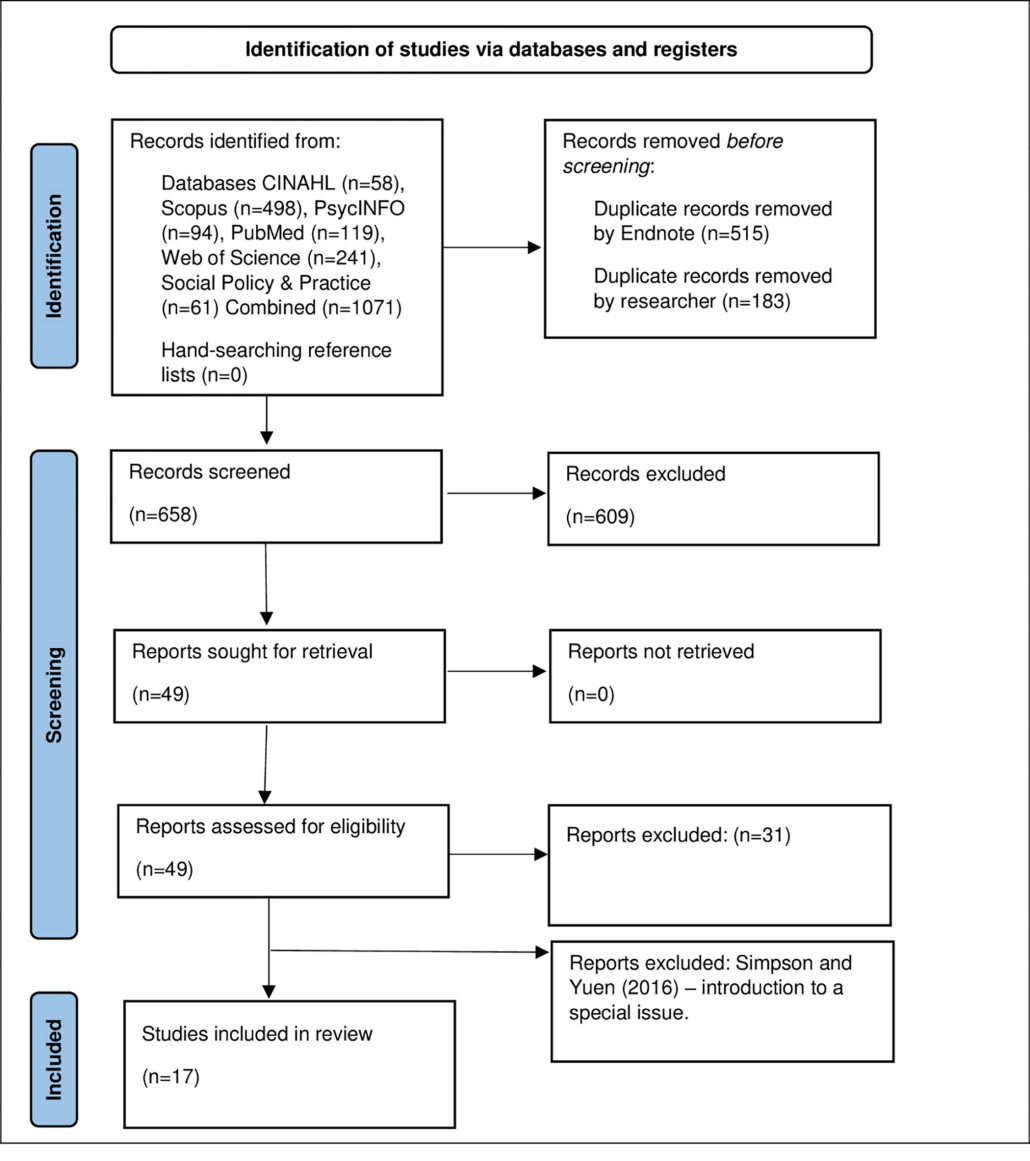
PRISMA flowchart.

Records were exported to Endnote [28] and reviewed in Rayyan [29]. Electronic searches identified 1071 records from CINAHL (n = 58), Scopus (n = 498) PsycINFO (n = 94), PubMed (n = 119), Social Policy & Practice (n = 61) and Web of Science (n = 241). Duplicates (n = 183) were removed with the remaining articles (n = 658) imported into Rayyan [29] for review. Working in pairs, four authors reviewed the titles and abstracts of all articles and met to discuss decisions around inclusion/exclusion. Authors excluded 609 articles at this stage and retrieved 49 for full text review. After applying the inclusion/exclusion criteria and once again meeting to discuss, 31 further articles were excluded, leaving 18 for inclusion in this review. Once data extraction had commenced it became apparent that one further paper [30] should be excluded due to it being an editorial which described the work of other authors in a special issue. The complete selection process is described in Fig 1 in accordance with PRISMA guidance [25].

### Data extraction

Standardised data extraction tools were created for the purposes of this review. Data were independently extracted by two reviewers with a third available to arbitrate disagreements. For empirical articles, including case studies, the extracted data comprised author name, date, aim, design, participants, measures and key findings. Results of this data extraction can be found in Table 2.

**Table 2:** Characteristics of Included studies; research papers.

Author(s), Country of Origin, Type of Brain Injury	Aim(s)/ Hypothesis	Design	Participants	Methods	Findings	Class of Evidence & Quality Appraisal (MMAT)
Conrick et al., (2022) USA TBI	Evaluate the association between years of social work practice, or self-reported participation in TBI-specific trainings, and TBI- related knowledge and confidence in serving clients with TBI.	Cross sectional survey.	834 Social Workers (female n = 704, male n = 115, non-binary n = 7, trans n = 2, unknown n = 6).	Survey created from a literature review, clinical and research experts, which gathered data on interactions, challenges and confidence in working with clients with TBI, knowledge and beliefs about TBI, training experiences and recommendations.	Training provided for social workers is insufficient to meet expected standards when working with clients. Social work students should be equipped with the knowledge and skills to support clients, advocate for their needs, recognise the disparities in TBI, comorbid conditions and distinguishing TBI symptoms from other conditions. Authors drew attention to impact of socioeconomic factors affecting clients (e.g. lack of insurance) and issues with coordinating care across multidisciplinary teams.	Class III Evidence Medium Quality
Coxe et al., (2021) USA TBI	To examine current social work practices in providing care to clients with co-occurring TBI and Substance Use Disorder (SUD).	Qualitative semi-structured interviews	17 Social Workers (female n = 13, male n = 4).	Semi-structured interview guide was developed based on existing literature regarding practice patterns of case managers providing services to TBI survivors [73] and professionals’ knowledge and misconceptions about TBI [74].	Insurance restrictions and lack of disability supports were barriers to care. Themes included: lack of basic knowledge about TBI and multiple roles in serving adults with co-occurring TBI and SUD. Recommendations included building workforce capacity, improving interpersonal relationships, addressing current policy and education, strengthening community partnerships, and addressing systemic and structural changes.	Class III Evidence High Quality
Jellema et al., (2021) Netherlands ABI	To understand how the social networks of people with ABI facilitates or hinders resumption of their activities, and how this affects their well-being and quality of life.	Qualitative	41 social workers (male n = 9), ages 22–64, educated to bachelors (n = 40) or masters (n = 1) in social work, psychology or behavioural sciences.	Narratives were collected from social workers during writing sessions where each provided cases in which a patient was a) facilitated and b) hindered by their social network to resume an activity.	Findings related to: availability of social network members (physically and emotionally); acknowledgment of patient’s possibilities and skills; respect for wishes, concerns and desires; inclusion in activities; activity enablement; encouragement; skill development.	Class III Evidence High Quality
Moore et al., (2014) USA mTBI (mild TBI)	To determine acceptability and preliminary effectiveness of the Social Work Intervention for Mild Traumatic Brain Injury (SWIFT-Acute) intervention on alcohol use, community functioning, depression, anxiety, post-concussive symptoms, post-traumatic stress disorder and service use.	Cohort study	Intervention Group: 32 Individuals with Brain Injury (69% male).Control Group: 32 individuals with brain injury (78% male),	Short Orientation Memory and Concentration Test (SOMCT); Alcohol Use Disorders Identification Test (AUDIT), the Community Integration Questionnaire (CIQ), qualitative patient accept-ability survey, Patient Health Questionnaire-4 (PHQ-4), Rivermead Post-concussion Symptoms Questionnaire (RPQ), Post-traumatic Stress Disorder Checklist-Civilian (PCL-C) and service use survey.	SWIFT-Acute was acceptable to patients. There is preliminary evidence of effectiveness for reducing alcohol use and preventing functional decline.	Class II Evidence (Prospective, non-randomized cohort study) High Quality
Moretti et al., (2017) Italy sABI (severe ABI)	Present the outcomes of interventions to identify a support path which stimulates social workers to reconsider some of the ways in which they work.	Qualitative.	18 patient families, 15 male, ages 28–76 (66.6% aged 41–60)	Interviews with patients and families, two focus groups with social workers. SWs recorded all activities undertaken in a weekly diary, including the people contacted and type of action, and content of interviews.	Importance of integrated social and health care for the person and their family, and the development of a functional network of support. Importance of training of individuals helping families, in order to identify appropriate interventions and collaboration between operators.	Class III Evidence Medium Quality
Simpson et al., (2016) Australia TBI	To examine the balance of person-centred versus environment-centred interventions delivered by social work in comparison to these other published interventions.	Quantitative longitudinal	27 family members (male n = 10, female n = 17, parents n = 13, spouse n = 9, adult child n = 2, adult sibling n = 3), average age 49 (SD 9)	Two coding systems: The Allied Health Activity Codes and the Indicators For Intervention (IFIs) developed by the National Allied Health Casemix Committee	The amount of indirect service required for the person-centred interventions was negligible. Work on environment-centred interventions comprised indirect hours (direct hours = 6.0 ± 6.0; indirect hours = 5.0 ± 6.0), involving the liaison or advocacy with service providers and service systems on behalf of the family. Three major areas of social work activity were identified: education and information, counselling, and case management (predominantly discharge planning).	Class III Evidence Medium Quality
Vungkhanching & Tonsing (2016) USA TBI	To examine the relationships between role clarity, workplace stress, perceived respect, value of self, and team collaboration among social workers working in an interdisciplinary team in a brain injury setting.	Quantitative survey	37 Social Workers (female n = 32, male n = 5), ages 25–34 (n = 7), 35–44 (n = 10), 45–54 (n = 7), 55–64 (n = 10), 65+ (n = 3).	8-item Workplace Stress Scale Independent variables: role clarity questionnaire [75], Perceived Respect Scale, and Self-Esteem Scale, Collaborative Practice Assessment Tool, & demographic variables	Factors such as perceived respect from team members, value of self, and team collaboration were significantly associated with role clarity. Social workers had clear expectations about their role in the interdisciplinary team, and perceived themselves as a valued member. Care provided by members of interdisciplinary teams can be a very positive experience for the patients or consumers and their families.	Class III Evidence Medium Quality
Holloway & Tyrrell (2016) UK/Australia ABI	To illustrate how services can work to protect and support families, facilitating engagement with rehabilitation.	Case study	2 female parents with ABI	Descriptive case studies describing collaboration of multidisciplinary teams to support individuals to parent effectively.	ABI requires a proactive approach to engage and support families and not simply a reactive, crisis-driven, and safeguarding-led one. Understanding the functional and emotional impact of ABI (what works to support sustained engagement, rehabilitation, and adaptation), as well as the child’s needs, social workers can work alongside clients to support not only parenting, but also change and adaptation. Integrating knowledge from multiple professionals will better equip SWs to prevent abuse.	Class III Evidence High Quality
Holloway & Fyson (2016) UK ABI	Rectify the knowledge deficit of social workers by providing information about ABI and discussing some of the challenges which social workers may face, particularly in the context of personalisation.	Case study	3 individuals with TBI (male n = 2), causes of TBI = substance abuse, fall, RTA.	Case study description of individuals’ situation	Effective and accurate assessments of need following ABI require specialist knowledge. Assessment should be timely following injury, take place over time and involve sufficient face-to-face contact with the individual and their social networks, in order to build trust. Personalised practice is essential to good outcomes.	Class III Evidence Medium Quality
Raju et al., (2016) India TBI	To provide basic understanding and suggest psychosocial interventions that can be provided for TBI caregivers during hospitalization in emergency and trauma care from medical and psychiatric social work (MPSW) perspective.	Case report	1 male caregiver aged 34, (patient wife female, aged 28)	None, description of input from MPSWs during hospitalization following TBI.	Support systems are inadequate and the psychosocial needs of survivors of TBI and their caregivers are overlooked. Simple explanation of psychological consequences of trauma and improving self-efficacy helps patients and family members to handle trauma reactions better. Providing adequate social support for caregivers during hospitalization moderates distress, stress, and anxiety.	Class III Evidence Medium Quality
Wurr (2012) UK ABI	To highlight the challenges that case managers face in accessing appropriate statutory services and funding for young adults with ABI.	Case study	1 female participant with ABI	None	For many people who ABI after maturation, a learning disability team would not be appropriate. In such cases they may be referred to physical disability teams who may lack understanding of their cognitive needs or mental health teams who may lack understanding of their physical needs. Here, services failed to recognise the patient’s main presenting need as the effects of ABI. She was assessed incorrectly (focusing on her physical disabilities) which resulted in failure to assess her cognitive functioning and her mental capacity under the Mental Capacity Act (2005) to make specific decisions regarding future accommodation.	Class III Evidence Medium Quality

The following data were also extracted from review articles; authors name, country of origin, type of ABI, aim, review design, included studies, inclusion criteria and findings. Extraction of information from included studies comprising expert opinion and guidance included: authors name, country of origin, type of ABI, aim, design, key discussion points. Results of the extracted data can be found in Tables 3 for review articles and 4 for expert opinion.

**Table 3:** Characteristics of included studies: Reviews.

Author(s), Country of Origin, Type of Brain Injury	Aim(s)	Design	Included Studies	Inclusion Criteria	Findings	Quality Appraisal (JBI Critical Appraisal Checklist for Systematic Reviews and Research Syntheses)
Holloway & Norman (2022) UK ABI	To review Safeguarding Adult Reviews (SARs) pertaining to individuals with ABI since 2014.	Literature review, thematic analysis	Six SARs included in the review, all adult male patients with ABI.	Inclusion criteria: SARs published in the UK since the ‘Tom’ (2014) case, that included individuals who had either been formally identified as having aa ABI within the review or had reference to medical history that would indicate a brain injury.	A lack of awareness of the needs of those with ABI and their families, and around the symptoms and nuances of ABI, particularly executive impairment and mental capacity, among social workers. Poor interdisciplinarity led to a lack of shared communication and decision-making with knowledgeable professionals. Poor understanding of aspects of the mental capacity legislation, particularly around unwise decisions, led to inappropriate or absent mental capacity assessments. Lack of professional curiosity led to a lack of action where intervention or assessment was required.	Medium Quality
Mantell et al., (2018) UK TBI	To identify the output, impact and quality of publications authored by social workers on TBI.	Scoping review	115 items were published that met the search criteria (intervention studies n = 10; observational studies n = 52; literature reviews n = 6; expert opinion or policy analysis n = 39; and others n = 8).	Papers were included if they were a chapter, book or article published in peer reviewed journals, focused on TBI, participants were aged 18–65, authored or co-authored by a Social Worker, published between 1970 and 2014.	Future research should seek to increase the number of controlled or cohort studies. Papers published in rehabilitation journals were cited more highly than those in social work journals. Adopting an academic–practitioner model with a focus on practice-research can ensure that future work is clinically relevant, with a focus on developing and evaluating interventions, while also providing pathways for building research capacity among social workers.	High Quality

**Table 4:** Characteristics of Included Studies: Discussion/Opinion/Practical Guidance publications.

Author(s), Country of Origin, Type of Brain Injury	Aim(s)	Design	Key Discussion Points	Quality Appraisal (JBI Critical Appraisal Checklist for Text and Opinion Papers)
Brain Injury Social Work Group & British Association of Social Workers (2016) UK ABI	To increase awareness of ABI among social workers and to provide guidance about what an ABI is and how intervention by social workers can benefit individuals.	Guide for social workers based on guidance from the Professional Capabilities Framework (PCF) and the Knowledge and Skills Statement for adults and child and family social work.	Guidance defines ABI, describes problems cause by it, and describes process of assessment and post-assessment support. Highlights key welfare benefits available after ABI.	Medium Quality
Brain Injury Social Work Group & British Association of Social Workers (2019) UK ABI	To increase awareness of ABI among social workers and provide guidance about what an ABI is and how social work intervention can benefit these individuals.	Guidance document.	Good social work practice depends on building trusting relationships between individuals and social workers. Local authorities must ensure that any adult who appears to have care and support needs, and any carer who appears to need support, receives a proportionate assessment which identifies their level of needs. If someone lacks capacity, they must be seen face to face. It is important not to miss the impact of a brain injury in the assessment process. Assessments may be time consuming, social workers should allow extra time for people with ABI. Working in partnership with the health service, local authorities, and charities is essential for SWs when assessing people with ABI. Assessments should be holistic, include an account of individual’s wishes and the views of their support network, be transparent, be conducted from a strengths-based perspective with a view to positive risk taking. Social workers need to equip themselves with skills, support and guidance that support effective communication.	High Quality
Degeneffe (2016) USA ABI	To raise awareness of the need to apply professional attention in research, clinical practice, and education to the neglected population of siblings of persons with ABI.	Discussion piece	Social work researchers should expand our limited understanding of sibling response to ABI. Social-work-based research should partner with scholars from academic disciplines engaged in sibling and ABI family research. Further examination on the life-span impact of ABI on siblings is required. Areas needing advocacy in the US include fully funding the TBI Act, increasing TBI Medicaid Waiver Programs, and motivating the Rehabilitation Services Administration to provide training on vocational rehabilitation for persons with ABI. Social workers should tailor their interventions to meet the specific support needs of younger versus older populations.	Medium Quality
Moore (2013) USA mTBI (mild)	Provides a summary of mTBI and its epidemiology, an overview of outcomes after mTBI in both civilian and military populations, and the state of research on psychosocial interventions for mTBI. Guidelines for current research and practice are highlighted.	Discussion piece	Improving understanding of mTBI and building empirical evidence for social service interventions after mTBI have important patient care implications. Gaining a clear picture of the needs of this population through practitioner and researcher collaboration is required to achieve these goals. Social Workers can encourage medical providers to screen for mTBI and frequently re-evaluate symptoms and coping strategies. Social work researchers can play an important role in improving patient care by collaborating with social work practitioners to understand mTBI symptom course, and developing and testing social work interventions. Recommendations include more research on the effect of targeted early patient interventions, and provision of information for patients about mTBI symptoms, coping strategies and reassurance about recovery.	High Quality

### Quality assessment

Reviewers employed the Mixed Methods Assessment Tool (MMAT) [31] to determine the quality of included empirical studies. The MMAT asks seven questions about qualitative and quantitative methodologies with studies then being rated as having low, medium of high levels of quality [31]. Ratings on each of the empirical studies, together with a description of the reasons for these, are included in Table 5 in accordance with MMAT guidance [31]. The Joanna Briggs Institute (JBI) checklists for systematic reviews and research synthesis [32] (see Table 6), case reports [33] (see Table 7) and text and opinion [34] (see Table 8) were used to assess all other included articles. Studies were independently assessed by two reviewers with a third available in the event of disagreement. Consensus was reached for decisions on all included articles.

**Table 5:** Quality assessment of empirical studies using the MMAT.

First Author & Year	Q1	Q2	Q3	Q4	Q5	Explanation
Conrick (2022)	Y	N	N	N	Y	Sample is not representative of the target population, measurements are not appropriate and risk of nonresponse bias is not low.
Coxe (2021)	Y	Y	Y	Y	Y	All criteria clearly addressed
Jellema (2021)	Y	Y	Y	Y	Y	All criteria clearly addressed
Moore (2014)	Y	Y	Y	Y	Y	All criteria clearly addressed
Moretti (2017)	Y	Y	Y	CT	CT	Interpretation of results may not be sufficiently substantiated by data and not enough evidence is provided to see coherence between qualitative data sources, collection, analysis and interpretation.
Simpson (2016)	Y	Y	Y	CT	Y	No comment is provided on nonresponse bias.
Vungkhanching (2016)	Y	Y	Y	CT	Y	No comment is provided on nonresponse bias.

(Y = YES–the paper clearly addresses the question, N = NO–the paper has clearly shown a negative answer to the question, CT = Cannot Tell–no statement is included which allows an answer to be drawn for the question***)*** Quality Assessment scoring: High: Y = 4–5, Medium: Y = 2–3, Low: Y = 0–1

**Table 6:** Quality assessment: JBI Checklist for systematic reviews.

First Author & Year	Q1	Q2	Q3	Q4	Q5	Q6	Q7	Q8	Q9	Q10	Q11	Explanation
Holloway & Norman (2022)	Y	Y	Y	U	U	Y	N	Y	U	Y	Y	Unclear if sources used to search for studies were adequate, and if the criteria for appraising studies was appropriate. Methods were not used to minimize errors in data extraction and the likelihood of publication bias was not assessed.
Mantell (2018)	Y	Y	Y	Y	Y	Y	Y	N/A	N	N	Y	Likelihood of publication bias was not assessed, and recommendations for policy and/or practice were not supported by the reported data.

(Y = YES–the paper clearly addresses the question, N = NO–the paper has clearly shown a negative answer to the question, U = Unclear–no statement is included which allows an answer to be drawn for the question, N/A = Not Applicable) Quality Assessment: High Quality: Y = 8–11, Medium Quality: Y = 5–7, Low Quality: Y = 0–4

**Table 7:** Quality assessment JBI checklist for case reports.

First Author & Year	Q1	Q2	Q3	Q4	Q5	Q6	Q7	Q8	Explanation
Holloway & Tyrrell (2016)	Y	Y	Y	Y	Y	N/A	N/A	Y	All criteria clearly addressed
Holloway & Fyson (2016)	Y	N	Y	N	N	N	N	Y	Patient history, diagnostic tests or assessment methods, intervention, post-intervention clinical condition and adverse events were not described.
Raju (2016)	Y	N	Y	N	Y	N	N	Y	Patient history, diagnostic tests or assessment methods, post-intervention clinical condition and adverse events were not described.
Wurr (2012)	Y	N	Y	N	N	N	N	Y	Patient history, diagnostic tests or assessment methods, intervention, post-intervention clinical condition and adverse events were not described.

(Y = YES–the paper clearly addresses the question, N = NO–the paper has clearly shown a negative answer to the question, N/A = Not Applicable) Quality Assessment: High: Y = 6–8, Medium: Y = 3–5, Low: Y = 0–2

**Table 8:** Quality assessment: JBI checklist for text and opinion papers.

First Author & Year	Q1	Q2	Q3	Q4	Q5	Q6	Explanation
Brain Injury Social Work Group (2016)	Y	Y	Y	N	N	N/A	The stated position is not the result of an analytical process and there is no reference to the extant literature.
Brain Injury Social Work Group (2019)	Y	Y	Y	N	N	N/A	Position is not the result of an analytical process, there is no reference to the extant literature.
Degeneffe (2016)	Y	Y	Y	N	Y	Y	The stated opinion is not the result of an analytical process.
Moore (2013)	Y	Y	Y	Y	Y	U	Author does not discuss any incongruence with the literature/sources clearly.

(Y = YES–the paper clearly addresses the question, N = NO–the paper has clearly shown a negative answer to the question, U = Unclear–no statement is included which allows an answer to be drawn for the question, N/A = Not Applicable) Quality Assessment: High quality: Y = 5–6, Medium quality: Y = 3–4, Low quality: Y = 0–2

We also sought to determine the classification of evidence of included studies as a measure of strength of the evidence provided. Reviewers judged each included study as providing Class I, II, or III evidence [35, 36] Class III evidence comprised case studies or clinical series without concurrent controls. Class II evidence included prospective, non-randomized cohort studies, retrospective, non-randomized case control studies or clinical series with well-designed controls. Lastly, Class I evidence included studies which utilised prospective, properly designed, randomized controlled trials (RCTS).

### Data analysis

A narrative synthesis [37] approach was employed to analyse the data due to the broad scope of included studies. A narrative synthesis relies on a textual rather than a statistical approach to combine the findings from included studies to tell the story of why a particular approach, policy or intervention does or does not work [37]. Taking a narrative synthesis approach allowed us to develop themes which were common across similar papers to address our review question.

## Results

### Study characteristics

A total of 17 publications were included in the final review, including research papers (n = 7), case studies (n = 4), discussion/opinion/practical guidance (n = 4) and literature reviews (n = 2). Further details on included publications can be found in the Characteristics of Included Studies tables (Tables 3–5). Included publications originated from six countries, with the UK and USA being the most common (n = 7 and n = 6, respectively) with remaining papers coming from the Netherlands [38], Italy [39], Australia [40] and India [41]. Of the included research papers, 4 were quantitative [40, 42–44] and 3 were qualitative [38, 39, 45]. Research papers included a range of 17–834 participants and ages ranged from 22–76 years.

### Quality appraisal

Following current guidance for the four quality assessment tools used in this review, included publications were assessed as high (n = 9) and medium (n = 8) methodological quality. A description of each included papers’ quality can be found in Tables 5–8. The Mixed Methods Appraisal Tool (MMAT) [31] was used for the 7 included research papers, five of which were rated as high quality, while the remaining two were rated as medium quality; Conrick et al [44] did not recruit a sample that was representative of the target population and did not use appropriate measurements for the aims of the study; Moretti [39] had not substantiated results with sufficient data. The four included case reports were assessed using the Joanna Briggs Institute (JBI) Checklist for Case Reports [33]. One case report [15] was assessed as high quality, while the remaining reports [41, 46, 47] did not describe patient history, diagnostic tests or assessments, interventions, or adverse events. The four included text and opinion papers were assessed using JBI Checklist for Text and Opinion [34], two of which [42, 48] were assessed as high quality, while the remaining were assessed as medium quality for the following reasons: Brain Injury Social Work Group [49] and [50] did not make reference to the extant literature and the stated position was not the result of an analytical process. The two remaining literature reviews were assessed for quality using the JBI Checklist for Systematic Reviews and Research Syntheses [32], and were assessed as high quality [20] and medium quality [21] as the criteria for assessing studies was not clearly described, and methods were not employed to minimise errors in data extraction.

Included research papers and case reports were also assessed for classification of study design, with each paper being rated as Class III evidence, whereas one [51] was rated as Class II evidence as a prospective, non-randomized cohort study. This draws attention to a need for an increase in numbers of cohort studies and potentially RCTs, as called for in the included scoping review by Mantell et al [20].

### Narrative syntheses

Following narrative synthesis of included publications four themes emerged from the data. These related to 1. ‘Advocacy and Social Work’—social workers’ responsibility to advocate for people with ABI and to encourage self-advocacy; 2. ‘Training and multidisciplinary team working’—work force planning for social workers in terms of training needs, role expectations and assessment guidelines; 3. ‘Inclusion of Social Networks’—the importance of social networks and inclusion of family members in supporting people with ABI; and 4. ‘Societal Barriers’—societal barriers that inhibit individuals from accessing appropriate support.

### Advocacy and social work

This theme related to the role of social workers in adequately advocating for people with ABI and their responsibility to encourage these individuals to develop their own skills in self-advocacy. In qualitative interviews conducted by Coxe et al [45], social workers identified one of their primary roles as being that of advocacy. This was also recognised in several publications [15, 38, 40, 41, 44, 45, 47, 48, 50], which highlighted the importance of ensuring that social workers are trained to adequately advocate for people with ABI. In UK guidance published for social workers, recognition was given to the responsibility of the social worker to holistically conduct assessments of need from a person-first, strengths-based perspective, in order to advocate appropriately for their needs [50] (50). In the UK and Australia, both Holloway and Fyson [47]and Simpson et al [40] recognised the importance of advocacy and taking the individual’s needs and abilities into consideration during assessments [47], while Degeneffe [48]drew attention to how social workers should use their advocacy skills to improve community-based services in the USA.

An extension of the theme of advocacy was that of self-advocacy and self-efficacy. According to Jellema et al [38], these are skills that social workers should be supporting in people with ABI to develop, in order to improve their capacity to resume activities following their injury. Raju et al [41] highlighted that by improving self-efficacy, patient reactions to trauma may be better managed through an explanation of what the person’s injury is and what the course of action may be. Holloway and Tyrrell [15], who conducted case studies in the UK and Australia, agreed that self-advocacy was also important when supporting parents with ABI to care for their children’s needs, and stated that social workers should draw on parents’ intrinsic motivation to care for their children to encourage advocacy.

### Training and multidisciplinary team working

A key area of concern amongst the included publications was the need for training of social workers engaged with people with ABI, in addition to collaboration between professionals. There is a need for training to be improved, as current practice results in social workers lacking basic knowledge of ABI and finding difficulty in recognising comorbidities in order to assign services to the individual [39, 44, 45]. As Moretti [39] suggests, adequate training would enable social workers to not only look for collaboration from multiple professionals when supporting people with ABI, but would also help to identify appropriate interventions for the individuals. Degeneffe [48] highlights that these interventions should be tailored to the age of the individual they are aimed at, identifying their specific support needs, and suggest that it is only through training that this can be achieved. Additionally, three publications [20, 21, 42] called for better collaboration of social work researchers and practitioners in order to develop and test appropriate interventions, improve knowledge about ABI and to ensure that future research is clinically relevant. Mantell et al [20] also highlighted that the current lack of collaboration has led to action not being taken in situations where interventions and assessment would have been beneficial. Furthermore, Holloway and Norman [21] highlight that training is needed on mental capacity legislation. In their review of safeguarding adult reviews, it was found that social workers were at risk of inadequately assessing mental capacity or failing to conduct the assessment altogether, due to a lack of training.

In order to better equip social workers to effectively support people with ABI, it is important to integrate knowledge from multi professional teams [15]. However, it is important that social workers have clear expectations of collaboration, to ensure effective team-work, and a clear understanding of what their role is in these teams [43]. A social worker’s role involves a variety of activities, as highlighted by Simpson et al [40] who found that the main areas of activity are in education and information for people with ABI, counselling, and case management, including assessments of need. These assessments are complex and thus should take place over time [47], allowing time for the involvement of multi professional teams [50] and must consider the individual’s cognitive functioning in order to assign appropriate ongoing support [46]. These recommendations should be recognised during training, and, if followed, will better prepare social workers to support people with ABI [49].

### Inclusion of social networks

Several publications raised awareness of the importance of an adequate and supportive social network for people with ABI [38, 39, 41, 47], in addition to how consideration should be given to the support needs of families of people with ABI [15, 21, 48]. Jellema et al [38] highlighted how individuals with ABI will experience an improvement in activity resumption if they have a supportive, encouraging network of people around them, who will enable the individual to participate in activities according to their potential, and be physically as well as emotionally available. This network should be comprised of individuals who are not health care professionals (i.e. family and friends), and social workers should be aware of the importance of this, encouraging and providing support to individuals to build this network while also providing support and guidance for caregivers who assume this role [39]. Social workers should ensure they allow sufficient time to meet face-to-face with individuals with ABI along with members of their social networks, in order to build trust and ensure that the social network are aware of the specific needs of the individual they are supporting [47]. Furthermore, Raju et al [41] notes that social workers should recognise that this social support is also important for caregivers, family and friends, to help them manage feelings of stress or anxiety about supporting their loved one following ABI.

Part of recognising the importance of social networks involves social workers considering the needs of family members in supporting individuals with ABI. Holloway and Norman [21] state that there is a distinct lack of awareness from social workers of the impact on the families of people with ABI, and what their support needs are. Training is needed for social workers to recognise these needs, however, training and education also needs to be provided for family members to ensure the best outcomes for their loved one, and to ensure families are equipped to manage difficulties involved in caring for the individual [39]. Research has shown the benefits of provding training on parenting skills [52], family-based problem solving [53], family-supported physical and cognitive rehabilitation [54] and behaviour management [55]. As stated in guidance for social workers [47 p.4], assessments of need should ‘take a whole family approach’, and social workers should adopt a proactive approach to providing support, rather than only acting if a safeguarding issue or crisis occurs [15]. As Moretti [39] highlights, social workers play a key role in recognising and working to resolve problems within the family and should be trained to do so in an effective and timely way. While social workers are provided with some training on dealing with families, Degeneffe [48] notes that further research and focus is needed on the support needs of siblings of people with ABI.

### Societal barriers

A final theme that emerged from this review, and that commonly emerges in reviews of this nature, focuses on the barriers in society that hinder individuals with ABI and their families from accessing necessary supports. In the USA, Coxe et al [45] found that insurance restrictions and a lack of provision of appropriate disability supports were resulting in people with ABI not receiving the care they needed. Furthermore, Conrick et al [44] also recognised the impact of socioeconomic factors on people with ABI, calling for current policy and systemic issues to be urgently addressed in order to access funding and accessible support for people with ABI. In India, Raju et al [41] also found a lack of disability support systems, and highlighted that social workers should aim to ensure that available resources are utilised to their fullest extent. In one of this review’s included case studies, Wurr [46] draws attention to the issues in the UK around identifying and accessing appropriate services, the costs involved, and the inadequate assessment of needs of individuals with ABI. Wurr [46] argues that disability services are inadequate and are failing individuals who require proper care, giving an example of an individual who was not assessed accurately and was therefore denied appropriate, supported accommodation that she desperately needed.

## Discussion

This review identified and synthesised research published between 2012 and 2022 on the topic of social workers and ABI. Articles included in this review were found to possess high (n-9) and medium (n = 8) levels of methodological quality as assessed by a number of standardised quality appraisal tools. However, when articles were classified according to the level of evidence they provided only one article [51] was classed as level II. Taken together, this indicates that while many of the existing studies were well conducted they were small-scale and did not provide strong evidence for their positions. The highest level of evidence as illustrated by our classification system refers to papers which are Class I and comprise well conducted, prospective RCTs. Others [20] have also noted the lack of rigorously conducted trials in the field of social work research. This may be due to a lack of suitable funding or an under appreciation for the need to provide high quality evidence when conducting new social work interventions. This may suggest that there are interventions, which have been introduced in the last ten years, that have not been adequately tested and which might be either ineffective or incapable of meeting the needs of survivors of ABI. It is necessary to ensure that any new interventions address the needs of those they purport to help and that there is clear evidence to the efficacy of these.

An important theme to emerge from this review was the need for social workers to act as advocates for their clients with ABI. In their online survey of 834 US based social workers, Conrick et al [44] identified the need for a range of advocacy tools to help social workers support positive outcomes for their clients. These authors acknowledge that many structural barriers exist for survivors of ABI which must be negotiated and challenged if survivors of ABI are to lead happy and fulfilled lives. In a qualitative study with 17 qualified social workers from the US, Coxe et al [45] showed that social workers believed that advocacy was particularly important for survivors of traumatic brain injury due to their possessing possible difficulties with communication and cognition. Social workers also felt that this advocacy role extended beyond health and social care to include raising public awareness of TBI [45]. A second element to social worker’s advocacy efforts related to the need to build the capacity of survivors of ABI to self-advocate. Social workers in Jellema et al’s [38] study felt it was imperative to provide a network for survivors of ABI through which they would resume activities, improve their quality of life and build self-esteem. Through this network survivors of ABI would gain the confidence to advocate for themselves [38]. Building the skills for survivors of ABI to become self-advocates has been investigated by Hawley [56] through the Self-Advocacy for Independent Life (SAIL) programme. This programme, developed through the Brain Injury Association of Colorado, comprised workshops and a workbook intended to improve self-advocacy beliefs, knowledge and behaviours. The programme was well received by participants and satisfaction was highly rated [56].

The second theme to emerge was the need for training on ABI for social workers and multidisciplinary working. It is a statutory requirement for social workers to practice safely and maintain their professional development [57]. Without provision of adequate training it is impossible for social workers to safely make decisions when working with survivors of brain injury. In some instances, clients may not be aware they have an ABI [58]. It is therefore important that social workers are capable of recognising the indicators of ABI so they may ask the right questions to explore a potential diagnosis and provide appropriate services. Examination of safeguarding adult reviews showed that social workers were at risk of inadequately assessing, or may fail to assess, mental capacity in survivors of brain injury due to a lack of training [21]. Holloway and Norman [21] also suggested that a lack of professional curiosity resulted in inaction on the part of social workers who were not aware of what interventions to employ. Training social workers on recognising the signs and symptoms of ABI (e.g. history of head trauma, loss of consciousness, hypoxia, behavioural deficits etc) is a crucial step in the provision of supportive services. Once an individual has been identified the expertise of the multidisciplinary team should be drawn upon to support social workers in identifying the most appropriate services that meet their clients’ identified needs. Social workers’ informed knowledge of ABI will, in turn, support the multidisciplinary team in making informed choices to better tailor interventions in supporting the health and social care needs of survivors of ABI. In addition to an identified training need, this review highlights the need for social workers to be aware of gaps in their knowledge and subsequently work within multidisciplinary teams. ABI is a complex condition which requires input from a variety of professionals skilled in their own fields.

Our review also showed the importance social workers ascribed to a supportive social network following ABI. We know that outcomes are improved for those with supportive networks and the incorporation of family and friends as part of the social workers role ensures that significant others are fully informed of the individual’s needs [59–61]. Social networks can grow and shrink over time with members becoming more or less important depending on the life stage of the individual. The social networks of survivors of ABI may contract due to physical, cognitive and social changes due to their injury. Researchers have suggested the importance of professionals’ reshaping their ideas of who constitutes family to extend beyond that of biological or judicial ties [62]. The social network of survivors of ABI are a key support which social networks should access in order to aid rehabilitation.

The last theme of this review identified the societal barriers which often hindered survivors of ABI in accessing the required services and supports. In regions where insurance restrictions dictated the services families could receive, it was noted that not all insurers covered all services and that social workers had to be cognisant of this fact and match clients to providers [45]. The erroneous assumption that ABI is a low incidence condition may contribute to the lack of funding invested into services [63, 64]. Further, ABI is a hidden disability which has no cure and therefore suffers from a lack of investment from medical and pharmaceutical companies. It is the responsibility of government agencies to provide adequate resource to support services for survivors of ABI with social workers having a clear role to play in lobbying for greater provision.

The social work profession faces mounting demands at a time when services are increasingly stretched. Research has shown that social workers are under increasing work pressures which impact on their well-being [65–67]. This has resulted in an increase in those choosing to leave the profession and high numbers of vacancies [68]. High turnover of social work staff has been shown to negatively impact on the relationship between the social worker and the service user [69] which ultimately leads to poor outcomes. Social workers have also experienced greater scrutiny of their role and an increasing administrative burden. A systematic review exploring social workers’ experiences of bureaucracy identified a number of important themes including deskilling the workforce, impact on personal well-being, losing sight of the client and their needs and resistance to bureaucracy [70]. However, social workers are committed to their work and gain satisfaction in helping to improve the lives of service users [71]. Social workers require continuing support for their well-being if they are to deliver high quality, effective practice which benefits the service users under their care.

### Suggestions for future research

This review found no Class I studies which examined social work-based interventions for people with ABI. Class I evidence refers to prospective RCTs and their absence suggests that no research has been conducted with social workers in this area. Further work should seek to develop and test interventions amongst social workers on how to better support people with ABI and their families. Interventions used in other professions (e.g. teaching) could be adapted to provide education and practical strategies for managing behaviour and providing accommodations for memory or executive functioning deficits [72]. Intervention strategies such as visual imagery, calendar use or mobile device reminders could be trialled in a real-world, social work context to provide scaffolding for clients with memory problems [42]. It would be important that any such development work includes the voices of people with ABI and their families in its design and conduct to ensure the interventions adequately address their needs.

The relatively small number (n = 11) of empirical studies included in this review which focused on social workers and ABI would suggest a lack of focus from the research community on this topic. Together with a lack of training it would suggest that the majority of social workers possess limited understanding of ABI. However, no studies have sought to explore the understanding of social workers on ABI in the way this has been conducted in other professions (education, nursing, medicine). It would be important to assess such understanding to determine where gaps exist and identify areas where intervention is warranted.

Several of our included studies suggested the need for educational interventions to better train social workers in ABI. We suggest that an education programme could readily be created for inclusion in tertiary level and continuing professional education programmes to address this need. A subsequent piece of work would be the longitudinal monitoring of the uptake and effectiveness of any such educational programme in changing social work practice.

### Strengths and limitations of the review

Included studies in this review largely came from high income countries. Only one paper came from a low- or middle-income country (LMIC) which may suggest that we have missed a body of literature from these regions. This may also be due to our excluding studies published in languages other than English. Alternatively, it may mean that little research on social work and ABI is being conducted outside high income countries. A strength of our review is that we consulted with a specialist subject librarian in developing our search strategy. This helped ensure that we effectively interrogated the chosen electronic databases to locate relevant papers. We also sought to reduce bias in in this review by conducting multiple author data extraction and quality appraisal and used standardised tools to achieve this.

An important consideration for understanding this analysis and future implications is the international nature of our included studies. It is a strength of the review that we have drawn together evidence from across different countries and is interesting to note that themes span different contexts. It should be borne in mind, though, that these countries represent very different legal and practice contexts, hence the details of the implications of themes may vary and interventions to address them are likely to need to be adapted to fit.

## Conclusions

Social workers are key professionals who act as a conduit to service provision for survivors of brain injury. Some social workers have taken the time to upskill themselves about ABI, undertaking further education and training. However, the majority of the profession have not received training on ABI and are therefore not best placed to understand the importance of matching services to their clients’ needs. Without such training they are failing to practice safely which may result in reduced outcomes for their clients. It is crucial for all social workers to receive at least a basic level of training on ABI that is both evidence-based and effective.

## Supporting information

S1 Checklist (DOCX)
